# Proteomics as the final step in the functional metagenomics study of antimicrobial resistance

**DOI:** 10.3389/fmicb.2015.00172

**Published:** 2015-03-03

**Authors:** Fiona Fouhy, Catherine Stanton, Paul D. Cotter, Colin Hill, Fiona Walsh

**Affiliations:** ^1^Teagasc - Moorepark Food Research Centre, Fermoy, Ireland; ^2^Alimentary Pharmabiotic Centre, Cork, Ireland; ^3^School of Microbiology, University College Cork, Cork, Ireland; ^4^Department of Biology, National University of Ireland Maynooth, Maynooth, Ireland

**Keywords:** functional metagenomics, antibiotic resistance, proteins, microbiology, novel mechanisms

## Abstract

The majority of clinically applied antimicrobial agents are derived from natural products generated by soil microorganisms and therefore resistance is likely to be ubiquitous in such environments. This is supported by the fact that numerous clinically important resistance mechanisms are encoded within the genomes of such bacteria. Advances in genomic sequencing have enabled the *in silico* identification of putative resistance genes present in these microorganisms. However, it is not sufficient to rely on the identification of putative resistance genes, we must also determine if the resultant proteins confer a resistant phenotype. This will require an analysis pipeline that extends from the extraction of environmental DNA, to the identification and analysis of potential resistance genes and their resultant proteins and phenotypes. This review focuses on the application of functional metagenomics and proteomics to study antimicrobial resistance in diverse environments.

## INTRODUCTION

The discovery and development of antibiotics have been among the most significant successes in medical history. Globally, their application has saved millions of lives and significantly increased life expectancy. However, we now face the prospect of a post-antibiotic era, where the rate of bacterial antibiotic resistance acquisition and dissemination is far outpacing the development of novel, effective antibiotics (Davies and Davies, 2010). To limit the further spread of resistance to the point where all antibiotics are made redundant, there is a pressing need to identify and understand novel antibiotic resistance genes/mechanisms. Indeed, doing so could enable the discovery of novel antimicrobials and alternative therapies. In certain cases it may also enable detection and characterization of resistance genes before their dissemination.

## APPROACHES FOR STUDYING ANTIBIOTIC RESISTANCE GENES

Antibiotic resistance genes have been identified in diverse environments (Cantas et al., 2013) including soil (Allen et al., 2008), gull feces (Martiny et al., 2011) and, increasingly, in the human gut microbiota (Fouhy et al., 2014a,b). Scientists have applied a range of approaches to detect antibiotic resistance genes in environmental or non-clinical microbiomes, all of which have their own inherent strengths/limitations. While cultivation is the gold standard used to identify bacteria with acquired resistance mechanisms, this may be a complex issue in non-clinical environments (Walsh, 2013). Few studies have been performed to investigate the culturable resistome of soil, for example. One reason for the low number of studies investigating the culturable resistome of soil is that the lack of breakpoints or guidelines in this area prevents the absolute definition of resistance by minimum inhibitory concentration. Culture approaches have been used primarily to study the resistance gene complement in specific pathogenic bacteria (Nys et al., 2004), and are less suitable to studying the entire resistome of complex environments. Culture-based approaches benefit from their simplicity to execute and the low associated costs. Cultures from diverse microbiomes can be screened on antibiotic selective media to determine the presence and abundance of resistant isolates (Villedieu et al., 2003; Forsberg et al., 2012; Gijón et al., 2012; Walsh and Duffy, 2013). However, while such approaches may be useful as a first step in the investigation of resistance in an environment, further analysis is required, usually involving molecular and sequencing approaches to identify the resistance genes. Moreover, it has been well established that despite significant improvements in culturing techniques, at least 50–70% of microbes in an environment are not easily cultured (Eckburg et al., 2005). Thus, such approaches alone have a limited capacity to investigate the resistance of the unculturable proportion of samples.

Molecular techniques typically complement culture-based studies (Walsh and Duffy, 2013). Such approaches include PCR-based detection of antibiotic resistance genes. Multiplex approaches can be applied to enable detection of a large number of antibiotic resistance genes simultaneously (Vakulenko et al., 2003; Looft et al., 2012; Popowska et al., 2012; Duffy et al., 2014). However, the major limitation lies in the requirement for some prior knowledge of the resistance genes being investigated to enable primer design. Thus, PCR-based approaches generally detect known resistance genes or genes sharing homology to known sequences. Despite these limitations, PCR-based approaches have been successfully applied to study antibiotic resistance. In one instance, a study of antibiotic resistance amongst members of a remote Indian community with minimal antibiotic exposure used PCR approaches to identify resistance genes, as well as integrons involved in gene mobilization (Pallecchi et al., 2007). In other cases, PCR-based approaches have been used to study the dissemination of particular resistance genes amongst bacterial groups (e.g., through identification of integrons or plasmid-encoded resistance (Kobayashi et al., 2013), such as that seen in extended spectrum β-lactamase (ESBL)-producing *Enterobacteriaceae* (Gijón et al., 2012).

## FUNCTIONAL METAGENOMICS

While culture- and PCR-based approaches have successfully identified resistance genes, metagenomic approaches have greatly enhanced our ability to study the entire resistome, rather than targeting just one bacterial species or culturable bacteria. Sequence-based metagenomics involves sequencing fractionated DNA from the total microbiome fractionated DNA without prior culturing. This approach benefits from the extensive publically available sequence databases, with which the newly generated metagenomic sequence data can be compared. However, sequence-based screens facilitate the identification of putative resistance genes, but do not provide information on functionality. With the availability of diverse DNA sequencing platforms, which have lowered the cost and increased the speed and efficiency of sequencing, sequencing-based metagenomics is likely to be applied more extensively to antibiotic resistance detection and has already been used to successfully identify resistance genes in numerous animal microbiomes including, e.g., chicken caecum (Qu et al., 2008), cattle (Durso et al., 2011) and buffalo rumen (Singh et al., 2012). Functional metagenomics has been used frequently to detect novel antibiotic resistance genes in water and soil microbiomes (Torres-Cortés et al., 2011; McGarvey et al., 2012; López-Pérez et al., 2013; Amos et al., 2014; Su et al., 2014; Udikovic-Kolic et al., 2014).

**FIGURE 1:**
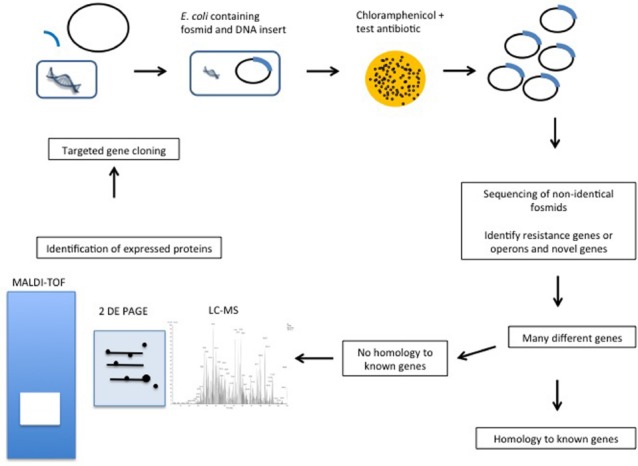
**Proposed functional metagenomics and proteomics combined gene function identification pipeline**.

Since the mid 1990s functional metagenomics has been used to screen metagenomic DNA in order to identify genes with specific functionalities (Healy et al., 1995; Handelsman et al., 1998). The approach is particularly valuable when screening for, or studying genes encoded by, microorganisms that cannot, or are difficult to, culture in the laboratory. Functional metagenomics relies on the cloning of DNA into plasmids (inserts of <10 kb), fosmids (25–45 kb inserts), cosmids (15–40 kb inserts), or BACs (bacterial artificial chromosome) (100–200 kb inserts) (Ekkers et al., 2012), expression of the genetic material in a suitable host and the utilization of a biological screen (frequently high-throughput) in order to identify the trait of interest (Figure 1). With regard to host, it is important to appreciate that the success of this approach relies upon the ability of the host to express the cloned metagenomic DNA. While additional surrogate hosts, including *Agrobacterium tumefaciens*, *Burkholderia graminis*, *Caulobacter vibrioides*, *Pseudomonas putida*, and *Ralstonia metallidurans*, have been developed for in-house functional metagenomic protocols, commercial kits rely on using *E. coli* as the host (Craig et al., 2010). After screening, one then needs to determine which of the, potentially many, cloned genes is responsible for conferring this phenotype. This is achieved through sequencing of the entire insert and *in silico* analysis (assuming the relevant gene is homologous to a gene of known function), mutagenesis of the vector (by transposon mutagenesis or other means) to select for lack of function and subsequent sequencing to identify the mutated gene, or sub cloning of the insert DNA and sequencing of sub clones that retain the activity of interest. Importantly, as a consequence of the screen being based on function alone, one can identify novel genes, i.e., it is not necessary to have a prior knowledge of gene sequence or origin of the gene, which are limitations of PCR- and culture-based approaches, respectively. Limitations of this technique include problems obtaining a representative sample of the microbial niche under investigation, successfully lysis all of the cells present, extracting DNA of sufficient quantity and quality to facilitate the creation of a DNA library or making a bank of sufficient size to ensure that it reflects the gene complement of the original community (Kowalchuk et al., 2007). The phenotype must also be due to the presence of a single gene, gene cassette or operon. Another key consideration relates to the choice of host into which the metagenomic DNA is cloned. In order for the screen to be successful, the relevant genes need to be expressed, mRNA be translated and the corresponding protein be functional, in this host (most frequently *E. coli,* though new alternatives continue to emerge; Liebl et al., 2014). Thus, different codon utilization, non-recognition of promoters, protein mis-folding, a missing capacity for cofactor synthesis or an inability to secrete/export the protein from the cell are just some of very many issues that can result in a relevant gene not being expressed in a heterologous host (Rosano and Ceccarelli, 2014). Studies are now coupling PCR of uncloned metagenomic DNA with functional screens to identify any resistance genes that may not have been captured, or were not expressed in the metagenomic library (Fouhy et al., 2014a). The success of the approach is also reliant on the phenotypic assay employed. Ideally, the assay should facilitate rapid screening, be specific in order to allow clear discrimination between positives and negatives and employ relatively inexpensive reagents/substrates. Despite these limitations, functional metagenomics has proved to be a powerful tool, enabling the identification and characterization of proteins of scientific and/or commercial interest from a vast variety of environmental niches that would otherwise be inaccessible.

## FUNCTIONAL METAGENOMICS TO IDENTIFY ANTIBIOTIC RESISTANCE GENES

It has been suggested that there may be more microorganisms in 1g of soil than there are humans on Earth (Trevors, 2010). It is thus not surprising that much of the early functional metagenomic work was completed with DNA sourced from soil. In addition, as approximately 80% of antibiotics in use today originated from soil bacteria it is not surprising that soil has been found to be a considerable resistance reservoir (Martin and Liras, 1989). Functional metagenomic studies on soil have identified novel resistance genes that provide protection against the major families of antibiotics (Allen et al., 2008; Torres-Cortés et al., 2011). Indeed, when McGarvey and colleagues screened over 1.4 million cloned genes against natural and synthetic antibiotics, they detected 39 unique cloned genes, with several sharing <40% amino acid sequence identity to their closest GenBank match (McGarvey et al., 2012). Similarly, a study of soil from an apple orchard treated with streptomycin also detected diverse novel resistance genes, including genes encoding aminoglycoside acetyltransferases, a multi-drug efflux pump, β-lactamases, and a novel bifunctional protein containing a fusion of a β-lactamase and a sigma factor (Donato et al., 2010).

Functional metagenomics is being increasingly applied to study the human gut microbiota as a reservoir of resistance. In one of the first functional metagenomic studies on this topic, Sommer et al. (2009) screened a functional library of a healthy adult gut microbiota with 13 antibiotics and identified 95 unique resistance-encoding inserts, including 27 unique β-lactamase sequences. Functional metagenomic studies have also shown the infant gut microbiota to be a resistance reservoir (Fouhy et al., 2014a), with the identification of β-lactam and aminoglycoside resistance genes. Moreover, Moore et al. (2013) successfully detected resistance against tigecycline; an antibiotic, which is not widely used. This suggests that functional metagenomics could potentially be used to screen for existing resistance to antibiotics undergoing development or for surveillance of resistance development.

Though functional metagenomics has demonstrated the ubiquitous nature of resistance genes, it is not always clear if specific genes pose a threat to human health. A recent study went some way to addressing this when the capacity of soil microorganisms to transfer resistance to human pathogens was investigated (Forsberg et al., 2012). The authors not only discovered novel resistance genes in the soil, but also highlighted recent exchanges between soil bacteria and clinical pathogens, as genes from non-pathogenic soil microbes contained identical genes to those present in human pathogens. The further application of these approaches will answer important questions regarding antibiotic resistance transfer and the clinical relevance of putative resistance genes detected using functional metagenomics.

## PROTEOMIC TOOLS IN BACTERIOLOGY AND UNDERSTANDING ANTIBIOTIC RESISTANCE

Transcriptomics identifies the transcripts present, i.e., the mRNA transcribed from the DNA, whereas proteomics identifies the proteins resulting from the translation of the transcripts into proteins. Proteomics has been used to analyze bacterial gene expression, but may also be used to identify post-translational modifications or protein turnover rates, which may be as important as the presence of the gene in the final bacterial phenotype (Gupta et al., 2007). Proteomics may utilize either gel-based or non-gel-based techniques. Microbial quantitative proteome analysis initially utilized two-dimensional gel electrophoresis (2-DE) followed by gel image analysis. The total cellular proteome of bacteria with differing resistance phenotypes could then be compared to identify proteins associated with an antibiotic resistance phenotype. The techniques evolved further to using a fluorescent labeling method called differential-in-gel-electrophoresis (DIGE) technique to identifying the differentially expressed proteins in two gels (Unlu et al., 1997). Following a wave of labeling technologies and gel-based techniques, the interfacing of mass spectrometry with liquid chromatography and improvements in this technology made the non-gel based methods competitive with the gel-based tools. Mass spectrometry allows for the direct quantitative comparison of different proteomes. Such proteomic tools have been used to directly investigate antibiotic resistance in specific pathogens and within bacterial populations and amongst human pathogens including *Salmonella typhimurium* (Coldham and Woodward, 2004) and *E. coli* (Xu et al., 2006) by comparing the proteins present in higher quantities in resistant bacteria in comparison to those in isogenic susceptible bacteria. The surface proteome and secretome of *Acinetobacter baumannii* was investigated under tetracycline stress (Yun et al., 2008). Although the transcript levels of major outer membrane proteins (OMPs) were not significantly changed, the proteomic analysis revealed that they were significantly decreased in response to tetracycline. Analysis of the secreted proteins indicated increased secretion of several OMPs under tetracycline stress. The increased secretion of particular OMPs was not due to transcriptional but rather to translational regulation, reflecting the necessity of using proteomics tools (Yun et al., 2008).

## COUPLING FUNCTIONAL METAGENOMICS WITH PROTEOMICS

Functional metagenomics offers significant promise to identify novel functionalities residing within the unculturable fraction of the human microbiome, as well as other complex niches. Much remains to be investigated, but the early results suggest that this will be vital and rewarding for future mining approaches. The steps from DNA cloning to identification of potential clones of interest can be performed in a high-throughput manner. However, there are limitations, in addition to those previously described that could benefit from the inclusion of proteomics in this process. The identification of the specific genes associated with a phenotype on a DNA insert that may be 50 kb in length, is particularly challenging. While DNA sequencing can provide the solution in some cases, if the insert sequence does not resemble known resistance genes then further investigations are required. The majority of these are low-throughput, thereby limiting the efficiency at which novel genes will be detected. One option is transposon mutagenesis. However, this usually functions by inactivating single genes and may not reveal other genes of relevance. Sub-cloning of the 50 kb insert can also provide a solution but can be time-consuming and doesn’t guarantee success, especially if more than one gene is required to create the phenotype. With high-throughput screening one may generate hundreds or thousands of clones of interest. Therefore individually cloning each section of each putative gene to test for the phenotype is neither efficient nor feasible. In addition, genes with no sequence similarity to known resistance genes but which confer a resistance phenotype are ignored. Similarly, if several genes are required for the resistance phenotype, they too would not be detected by functional metagenomics alone.

The use of protein analysis in combination with functional metagenomics could overcome these problems (Figure 1). Proteomics advances will enable us to investigate the proteins expressed in functional metagenomics, which would advance this technique further. Indeed, proteomics has been used to identify proteins involved in antibiotic resistance by comparing bacteria with and without a particular resistance phenotype (Pieper et al., 2006). In particular, 2-DE, polyacrylamide gel electrophoresis (PAGE) and matrix-assisted laser desorption/ionization time of flight mass spectrometry (MALDI-TOF) have been employed to study vancomycin resistance in *Staphylococcus aureus* (Pieper et al., 2006). With respect to functional metagenomics, by adding protein analysis one can identify which proteins are highly expressed in comparison to a control lacking the cloned DNA. The proteins that accumulate at high levels under the selective pressure, corresponding to those genes that are turned on in these conditions, can then be identified. It must also be noted that this is only accurate if the resistance gene responds only in response to the antibiotic and does not function under other circumstances, e.g., stress. There are certainly limitations in the analysis of proteins expressed but it is thought that this would identify a select number of genes from within the inserted DNA that are expressed and reduce the cloning required, e.g., 50 sub clones containing 1 kb inserts to perhaps five sub clones. The comparative quantitative proteome resulting from analysis using LC-MS would then identify the proteins at higher or lower levels in the bacteria, which are encoded in the same insert of the respective clone. If several proteins are required for the resistance phenotype they can all be identified. Therefore, novel processes may be identified rather than simply genes similar to those currently characterized as resistant.

## CONCLUSION

Functional metagenomics is now a component of the molecular toolbox used to investigate antibiotic resistance in complex microbiomes. This review suggests the addition of proteomics to this technique in order to strengthen the results generated.

### Conflict of Interest Statement

The authors declare that the research was conducted in the absence of any commercial or financial relationships that could be construed as a potential conflict of interest.
